# Genetic Background and Inbreeding Depression in Romosinuano Cattle Breed in Mexico

**DOI:** 10.3390/ani11020321

**Published:** 2021-01-28

**Authors:** Jorge Hidalgo, Alberto Cesarani, Andre Garcia, Pattarapol Sumreddee, Neon Larios, Enrico Mancin, José Guadalupe García, Rafael Núñez, Rodolfo Ramírez

**Affiliations:** 1 Department of Animal and Dairy Science, University of Georgia, Athens, GA 30602, USA; jh37900@uga.edu (J.H.); Alberto.Cesarani@uga.edu (A.C.); Andre.Garcia@uga.edu (A.G.); 2 Department of Livestock Development, Bureau of Biotechnology in Livestock Production, Pathum Thani 12000, Thailand; pattrapoljk@gmail.com; 3 Departamento de Zootecnia, Posgrado en Producción Animal, Universidad Autónoma Chapingo, Chapingo 56230, Mexico; larios.neon@gmail.com (N.L.); rafael.nunez@correo.chapingo.mx (R.N.); rrv33@hotmail.com (R.R.); 4 Department of Agronomy, Food, Natural Resources, Animals and Environment-DAFNAE, University of Padova, Viale dell’Università 16, 35020 Legnaro, Italy; enrico.mancin@phd.unipd.it

**Keywords:** autozygosity, effective population size, inbreeding, inbreeding depression, Romosinuano, runs of homozygosity

## Abstract

**Simple Summary:**

The objective of this study was to evaluate the genetic background and inbreeding depression in the Mexican Romosinuano cattle using pedigree and genomic information. Inbreeding was estimated using pedigree ($F_{PED}$) and genomic information based on the genomic relationship matrix ($F_{GRM}$) and runs of homozygosity ($F_{ROH}$). Linkage disequilibrium (LD) was evaluated using the correlation between pairs of loci, and the effective population size ($N_{e}$) was calculated based on LD and pedigree information. The pedigree file consisted of 4875 animals; 71 had genotypes. LD decreased with the increase in distance between markers, and $N_{e}$ estimated using genomic information decreased from 610 to 72 animals (from 109 to 1 generation ago), the $N_{e}$ estimated using pedigree information was 86.44. The number of runs of homozygosity per animal ranged between 18 and 102 segments with an average of 55. The average inbreeding was 2.98 ± 2.81, 2.98 ± 4.01, and 7.28 ± 3.68% for $F_{PED}$, $F_{GRM}$, and $F_{ROH}$, respectively. A 1% increase in inbreeding decreased birth weight by 0.103 kg and weaning weight by 0.685 kg. A strategy such as optimum genetic contributions to maximize selection response and manage the long-term genetic variability and inbreeding could lead to sustainable breeding programs for the Mexican Romosinuano cattle breed.

**Abstract:**

The ultimate goal of genetic selection is to improve genetic progress by increasing favorable alleles in the population. However, with selection, homozygosity, and potentially harmful recessive alleles can accumulate, deteriorating genetic variability and hampering continued genetic progress. Such potential adverse side effects of selection are of particular interest in populations with a small effective population size like the Romosinuano beef cattle in Mexico. The objective of this study was to evaluate the genetic background and inbreeding depression in Mexican Romosinuano cattle using pedigree and genomic information. Inbreeding was estimated using pedigree ($F_{PED}$) and genomic information based on the genomic relationship matrix ($F_{GRM}$) and runs of homozygosity ($F_{ROH}$) of different length classes. Linkage disequilibrium (LD) was evaluated using the correlation between pairs of loci, and the effective population size ($N_{e}$**)** was calculated based on LD and pedigree information. The pedigree file consisted of 4875 animals born between 1950 and 2019, of which 71 had genotypes. LD decreased with the increase in distance between markers, and $N_{e}$ estimated using genomic information decreased from 610 to 72 animals (from 109 to 1 generation ago), the $N_{e}$ estimated using pedigree information was 86.44. The reduction in effective population size implies the existence of genetic bottlenecks and the decline of genetic diversity due to the intensive use of few individuals as parents of the next generations. The number of runs of homozygosity per animal ranged between 18 and 102 segments with an average of 55. The shortest and longest segments were 1.0 and 36.0 Mb long, respectively, reflecting ancient and recent inbreeding. The average inbreeding was 2.98 ± 2.81, 2.98 ± 4.01, and 7.28 ± 3.68% for $F_{PED}$, $F_{GRM}$, and $F_{ROH}$, respectively. The correlation between $F_{PED}$ and $F_{GRM}$ was −0.25, and the correlations among $F_{PED}$ and $F_{ROH}$ of different length classes were low (from 0.16 to 0.31). The correlations between $F_{GRM}$ and $F_{ROH}$ of different length classes were moderate (from 0.44 to 0.58), indicating better agreement. A 1% increase in population inbreeding decreased birth weight by 0.103 kg and weaning weight by 0.685 kg. A strategy such as optimum genetic contributions to maximize selection response and manage the long-term genetic variability and inbreeding could lead to more sustainable breeding programs for the Mexican Romosinuano beef cattle breed.

## 1. Introduction

Creole cattle breeds in the American continent originated from the Iberian Peninsula cattle five centuries ago. One of them is the Romosinuano breed, developed from isolation and adaptation to harsh environments in Colombia and has spread mainly to Costa Rica, the United States of America, Venezuela, and Mexico [1]. Over time, the Creole cattle breeds have adapted to adverse tropical conditions; therefore, genes associated with extreme environments and parasite resistance have been selected by natural selection. In Mexico, the national herd of Romosinuano beef cattle was established with the use of germplasm from Costa Rica (Turrialba) and later from the United States (Florida State University) [1].

The Romosinuano breed captured the attention of Mexican beef producers because animals of this breed have good growth performance and meat quality, high fertility, and the ability to adapt to hot and humid conditions and large parasite infestation (e.g., ticks; De Alba [1]). In 1998, local breeders established the “*Asociación Mexicana de Criadores de Ganado Romosinuano y Lechero Tropical*” (AMCROLET; De Alba [1]). With the creation of AMCROLET, performance information started being recorded to establish a genetic evaluation to improve birth weight and weaning weights [2].

The national herd of Romosinuano beef cattle in Mexico is currently located in tropical areas in the Mexican states of Campeche, Michoacán, Tabasco, Tamaulipas, and Veracruz [2], and the existence of a large number of multiplier herds indicates that the national population is growing [3].

Núñez-Domínguez et al. [3] characterized the population structure and evaluated the genetic variability of the Mexican Romosinuano population with pedigree data and concluded that the genetic diversity has been decreasing, mainly due to random loss of genes. Hence, it is important to reduce such losses of genetic diversity to ensure the breeding program’s sustainability and the breed itself in Mexico. The accumulation of deleterious alleles due to the increase in inbreeding can reduce livestock’s performance and fitness. This reduction is referred to as inbreeding depression, and the study thereof is also crucial in populations with small effective population size.

The accuracy of genetic parameters and inbreeding coefficients estimated using pedigree information heavily depends on its integrity. For instance, pedigree completeness has a substantial effect on estimating the inbreeding coefficient because the probability of finding common ancestors increases with the degree of pedigree completeness [4] Furthermore, missing pedigrees lead to assumed relationships of zero among animals with and without pedigree information, which may not be true. To alleviate this problem, VanRaden [5] presented a method that substitutes these zero relationships by the average relationships among animals born in specific years. In the study by Núñez-Domínguez et al. [3] on the Romosinuano breed in Mexico, the pedigree completeness was low; for example, going back three generations, the pedigree completeness was only 65.2%. The authors highlighted the necessity of more and accurate data recording as well as better estimates of demographic and genetic parameters.

Recently, the development of molecular techniques and the availability of genomic information, such as high-density single-nucleotide polymorphisms (SNP) panels, have made it possible to expand genetic diversity studies in cattle populations. To explore the genetic diversity across the genome and develop tools to design sustainable and more appropriate breeding programs, the AMCROLET has recently obtained the genotypes of 71 animals (54 K SNP markers). The first objective of the present study was to evaluate the genetic background of the Romosinuano beef cattle breed in Mexico, using different approaches: (i) pedigree analysis; (ii); inbreeding coefficients estimated by pedigree and genomic information; (iii) analysis of linkage disequilibrium and effective population size. A second objective was to assess the effect of inbreeding on birth weight and weaning weight traits.

## 2. Materials and Methods

### 2.1. Pedigree Genotypes and Phenotypes

The AMCROLET provided the genealogical information, phenotypes, and genotypes used in this research study through the performance-recorded database for the Romosinuano beef cattle breed. The pedigree file consisted of 4875 animals, progeny of 219 sires and 1685 dams, born between 1950 and 2019.

Seventy-one animals were genotyped with the medium-density Affymetrix chip (54 K SNP markers). The genotyped animals were 22 males and 49 females born between 2003 and 2018 and were selected among the alive animals in the population-based on their contribution to the population (i.e., animals with more progeny were selected). The average number of progeny in the group of genotyped animals was 3.7 for males and 1.7 for females. Quality control on animals and markers was performed using PLINK software version 1.90 [6], according to the following parameters: minimum call rate equal to 95%, minor allele frequency of each marker greater than 1%, the threshold to exclude markers that deviate from Hardy–Weinberg equilibrium was a *p*-value set to 10^−6^. SNP mapped on sexual chromosomes or not mapped on the *Bos taurus* autosome (BTA) 3.1.1 release were also discarded. Markers mapped on sexual chromosomes were discarded due to big differences between males and females allosomes. Animals and markers that failed these quality control criteria were removed. All the animals were retained; a total of 30,571 SNP markers mapped on 29 autosomal chromosomes were retained for further analyses.

The phenotypic information included a total of 1328 birth weight phenotypes [mean (SD); 27.13 (4.88) kg], and 690 weaning weight phenotypes [mean (SD); 144.28 (27.92) kg]. Animals with phenotypes were born between 2001 and 2019 and had both sire and dam known. The number of genotyped animals with phenotypes were 51 for birth weight and 37 for weaning weight.

### 2.2. Pedigree Analyses

The pedigree analyses were performed using the Endog software version 4.8 [7]. The following parameters were calculated: pedigree-based inbreeding coefficient ($F_{PED}$), relatedness coefficient (AR), generation intervals, equivalent complete generations, realized effective population size, probabilities of gene origin, and the number of progeny per sire and dam. The pedigree analysis was done for the whole population (4875 animals), and a reference population consisted of 1058 animals born between 2013 and 2019. The reference population represents the animals born in the last generation of the population. It was defined based on the generation interval of this population estimated by Núñez-Domínguez et al. [3], which was ~7 years in this population.

#### 2.2.1. Inbreeding and Relatedness Coefficient

The pedigree-based inbreeding coefficient for each animal represents the probability that two alleles at any locus are identical by descent [8], and it was computed using the algorithm of Meuwissen and Luo [9]. Each animal’s relatedness coefficient was computed as the probability that an allele selected randomly from the entire population included in the pedigree belonged to a particular animal. The AR can be interpreted as the animal’s representation in the context of the entire pedigree, without knowledge of its pedigree [10].

#### 2.2.2. Equivalent Complete Generations

Equivalent complete generations assessed the completeness of the pedigree. Equivalent complete generations for an individual i ($E_{q}G_{i})$ were calculated according to Maignel et al. [11] as follows:

$$E_{q}G_{i}=\sum {\left(\frac{1}{2}\right)}^{n}$$

where n is the number of generations separating the individual from each known ancestor in the pedigree. The percentage of known ancestors during the last ten generations was calculated. The pedigree completeness index (PCI) was generated for each generation. The PCI represents the mean proportion of ancestors known in each ancestral generation and was computed as the proportion of known ancestors in each ascending generation. For example, the second generation for a given animal was assigned completeness measure 1.0 if all four grandparents were known, 0.75 if three were known, and so on [12].

#### 2.2.3. Generation Interval

The generation interval (GI) was calculated as the parents’ average age at their progeny’s birth time kept for reproduction. It was calculated across the four genetic pathways, sire of sire ($L_{ss}$), sire of dam ($L_{sd}$), dam of sire ($L_{ds}$), and dam of dam ($L_{dd}$). The average generation interval was computed as follows:

$$GI=\frac{L_{ss}+L_{sd}+L_{ds}+L_{dd}}{4}$$

#### 2.2.4. Realized Effective Population Size

The effective population size is the size of an ideal population, characterized by equal sex ratio, absence of mutation, migration and selection, which has the same inbreeding rate as the real population under study. The realized effective population size ($N_{e}$) was estimated based on the individual increase in inbreeding. The coefficients of individual increase in inbreeding ($\Delta F_{i}$) were computed according to the method described by Gutiérrez et al. [13] and modified by Gutiérrez et al. [14], using the following formula:

$$\Delta F_{i}=1-\sqrt[{E_{q}G_{i}-1}]{1-F_{i}}$$

where $F_{i}$ and $E_{q}G_{i}$ are the inbreeding coefficient and the equivalent complete generations for individual i, respectively. The coefficients of individual increase in inbreeding were averaged, and the realized effective population size was estimated as:

$$N_{e}=\frac{1}{2\bar{\Delta F}}$$

#### 2.2.5. Probabilities of Gene Origin

Changes in the genetic diversity and population structure, such as recent bottlenecks, were assessed based on the probability of gene origin. Two parameters on the probability of gene origin, including the effective number of founders and ancestors, were estimated. The effective number of founders ($f_{e}$) denotes the number of equally contributing founders that would result in the same level of genetic diversity in the current population, and it was estimated according to Lacy [15]:

$$f_{e}=\frac{1}{\sum_{k=1}^{m}q_{k}^{2}}$$

where $q_{k}$ is the expected proportional genetic contribution of founder k; computed as the average relationship of the respective founder to each animal in the population, and m is the total number of founders.

The effective number of ancestors ($f_{a}$) measures the minimum number of ancestors (not necessarily founders) explaining the complete genetic diversity of the current population, and it was computed according to Boichard et al. [10]:

$$f_{a}=\frac{1}{\sum_{k=1}^{n}p_{k}^{2}}$$

where $p_{k}$ is the marginal contribution of each ancestor, which is the contribution made by an ancestor not explained by a previously chosen ancestor, and n is the total number of ancestors.

The $f_{a}$ is lower than the $f_{e}$, and the comparison of both numbers can be used to evaluate the impact of bottlenecks that may have occurred from the founders to the present population [10]; the lower the $f_{a}$/$f_{e}$ ratio, the more stringent the bottlenecks were.

### 2.3. Genomic Analyses

#### 2.3.1. Inbreeding Coefficients

Individual genomic inbreeding coefficients were calculated by two methods. In the first method, genomic inbreeding coefficients were obtained from the genomic relationship matrix ($F_{GRM}$). The $F_{GRM}$ were calculated by subtracting one from the diagonal elements of the genomic relationship matrix (G), built according to VanRaden [16] as follows, and using the BLUPF90 program [17]:

$$G=\frac{ZZ^{\prime }}{2\sum p_{i}\left(1-p_{i}\right)}$$

where Z is the matrix of centered gene content, and $p_{i}$ is the minor allele frequency of $SNP_{i}$. The $F_{GRM}$ is sensitive to the allele frequencies used to compute the genomic relationship matrix [18,19]. Therefore, in this research study, allelic frequencies were fixed to 0.5 instead of calculated from the current genotypes because of the small number of genotyped animals.

In the second method, runs of homozygosity (ROH) were used to compute individual genomic inbreeding coefficients. Consecutive ROH were computed through the algorithm implemented in the R package “DetectRuns” [20]. Runs of homozygosity were defined as 15 consecutive homozygous SNP covering a least 1 Mb in length. To be more conservative, heterozygotes and missing markers were not allowed into ROH. The maximum distance between consecutive markers was 1 Mb. Average ROH length ($L_{ROH}$) was computed for each animal, and according to their length, ROH were grouped into five different classes: 1–2 Mb, 2–4 Mb, 4–8 Mb, 8–16 Mb, >16 Mb [21]. The number of ROH ($n_{ROH}$) and the sum of all the ROH ($S_{ROH}$) per individual were also computed. Specific ROH, i.e., homozygous regions starting and ending precisely at the same positions within the chromosome, were also computed [22]; these segments can be found in one or more animals. Individual ROH-based inbreeding coefficients were estimated at the genome-wide level ($F_{ROH}$) and by chromosome ($F_{ROH_{CHR}}$) as follows:

$$F_{ROH}=\frac{L_{ROH}}{L_{AUT}}$$

$$F_{ROH_{CHR}}=\frac{L_{ROH}}{L_{CHR}}$$

where $L_{ROH}$ is the total length of all the ROH detected in the animal’s autosomes, $L_{AUT}$ is the total length of the autosomal genome; calculated as the sum of the lengths of the 29 autosomal chromosomes (2507.77 Mb), $L_{CHR}$ is the total length of the chromosome, calculated as the length between the position of the first and the last SNP in the chromosome. The length of the chromosomes ranged from 42.90 Mb (BTA25) to 158.03 Mb (BTA1).

#### 2.3.2. Linkage Disequilibrium and Effective Population Size

The historical trajectory of effective population size was estimated through linkage disequilibrium (LD) using the SNeP V1.1 software [23]. The LD was evaluated using the squared Pearson’s product-moment correlation coefficient between pairs of loci ($r_{x,y}^{2})$ for unphased data as proposed by Barbato et al. [23] as follows:

$$r_{x,y}^{2}=\frac{{\left[\sum_{i=1}^{n}\left(Xi-\bar{X}\right)-\left(Yi-\bar{Y}\right)\right]}^{2}}{\sum_{i=1}^{n}{\left(Xi-\bar{X}\right)}^{2}\sum_{i=1}^{n}{\left(Yi-\bar{Y}\right)}^{2}}$$

where X and Y are two separate loci, $\bar{X}$ and $\bar{Y}$ are the mean genotype frequencies for the first and second locus, respectively, Xi is the genotype of individual i at the first locus, and Yi is the genotype of individual i at the second locus.

The trajectory of effective population size (Ne) was also estimated using the SNeP V1.1 software. This software estimates the historical Ne based on the relationship among $r^{2}$, Ne, and c (recombination rate), applying the following formula proposed by Corbin et al. [24]:

$$Ne\left(t\right)={\left[4f\left(c_{t}\right)\right]}^{-1}\left[E{\left(r_{adj}^{2}|c_{t}\right)}^{-1}-\alpha \right]$$

where Ne(t) is the effective population size t generations ago, which is equivalent to $t={\left[2f\left(c_{t}\right)\right]}^{-1}$, presented by Hayes et al. [25]; $f\left(c_{t}\right)$ is a mapping function related to the recombination rate; $r_{adj}^{2}$ is the LD adjusted for sampling bias. In the sampling bias adjustment (due to small sample size (n)), the input parameter is β in $r_{adj}^{2}=r^{2}-{\left(\beta *n\right)}^{-1}$, in this research study we used β=1 for unphased data. Because we studied long distances (>100 Mb) between SNP to estimate Ne in recent generations, we applied an adjustment to the recombination rate through the mapping function developed by Sved and Feldman [26] to translate the estimated linkage distance (d) into the recombination rate: $c=d\left(1-\frac{d}{2}\right)$. The value of α was 2 to correct for the occurrence of mutations as proposed by Tenesa et al. [27]. Other included options related to the minimum and maximum distance between SNP were *-mindist* (100 kb) and *-maxdist* (35,000 kb), respectively. Short distances allowed the estimation of Ne in distant generations, whereas longer distances allowed the estimation of Ne in recent generations.

### 2.4. Selection Signatures

Genomic regions under selection can be defined as highly homozygous (ROH islands) and heterozygous (ROHet islands) regions [28]. The ROH and ROHet islands were obtained based on the SNP frequency (times that each SNP was detected in a run divided by the number of animals) within ROH and ROHet, respectively. The SNP from the top 0.01% (99.9 percentile was defined as a threshold) of the distribution were selected to define a region as an “island”.

The runs of heterozygosity were obtained using the consecutive method in the R package “DetectRuns” [20]. The ROHet were defined as 15 consecutive heterozygous SNP covering at least 250 kb in length. Inside the ROHet, the allowed number of homozygous and missing markers was three and two, respectively. The maximum distance between consecutive markers was set to 1 Mb.

Identification of the genes within the highly homozygous and heterozygous genomic regions detected was obtained from the Genome Data Viewer tool provided by NCBI (https://www.ncbi.nlm.nih.gov/genome/gdv/browser/genome/?id=GCF_002263795.1). The *Bos taurus* genome assembly ARS-UCD1.2 was used as a reference.

### 2.5. Inbreeding Depression Analysis

Quality control on the phenotypes considered: (1) for both traits, observations outside of the range mean ± 3 standard deviations were discarded; (2) weaning weight phenotypes were adjusted to 240 d of age; animals with phenotypes recorded outside of the range 240 ± 45 d of age were removed.

Inbreeding depression was estimated for birth weight and weaning weight by regressing the phenotypes on inbreeding coefficients. The inbreeding depression was estimated in two groups of animals: (1) in all the animals with phenotypes and (2) in animals with phenotypes and genotypes. Pedigree-based inbreeding coefficients were used in both cases, while the inbreeding coefficients obtained by genomic analyses were used only in the second scenario. The traits were analyzed separately using the following linear model:

$$y_{ijk}=\mu +sex_{ij}+yob_{ik}+\beta_{1}F_{i}+e_{ijk}$$

where, $y_{ijk}$ is the phenotype of animal i belonging to the sex class j (j=1,2), born in the year k (k=1,2,…, 18), μ is the overall intercept, $\beta_{1}$ is the regression coefficient on the individual level of pedigree ($F_{i}=F_{PED}$) or genomic ($F_{i}=F_{GRM}\;or\;F_{ROH})\;$) inbreeding, and $e_{ijk}$ is the residual term assumed to be normally distributed.

All regression analyses and summary statistics were carried out using the R software [29].

## 3. Results and Discussion

### 3.1. Pedigree Analysis

In the population of Mexican Romosinuano beef cattle, the pedigree had 27% and 35% of missing information for sire and dam pathways, respectively. Therefore, it is necessary to improve the pedigree recording at the population level, especially from the maternal side. On average, the number of progeny was 16.24 ± 25.06 (maximum 144) per sire and 1.84 ± 1.72 (maximum 12) per dam. A total of 361 full sibs were found from the pedigree, representing 8% of all animals.

#### 3.1.1. Inbreeding and Relatedness Coefficient

The average $F_{PED}$ was 1.52% in the total population and 2.63% in the reference population. The percentage of inbred animals was 32.4% in the total population and 54.6% in the reference population. The average $F_{PED}$ was 4.69% in the inbred animals in the total population and 4.81% in the reference population. The $F_{PED}$ estimations in the total and reference populations suggest that the inbreeding level is low in the Mexican Romosinuano. However, underestimation due to the shallow and missing pedigrees must be recognized. Indeed, Barczak et al. [30] reported that a pedigree with many missing ancestors could lead to underestimated inbreeding coefficients. In the reference population, the percentage of inbred animals and the $F_{PED}$ were greater than that of the total population, indicating loss of genetic diversity that could adversely impact the animals’ performance adversely.

**Figure 1:**
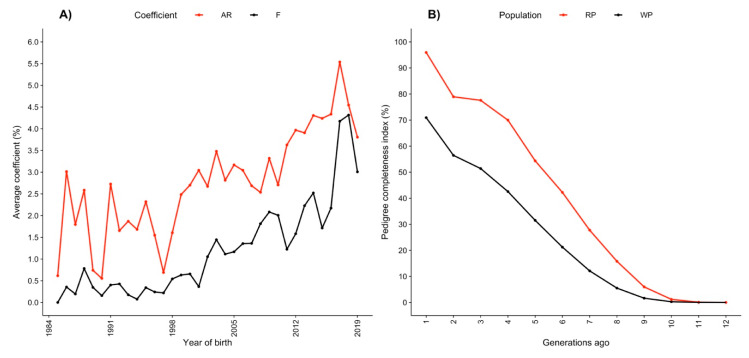
(**A**) Evolution of the average pedigree-based inbreeding ($F_{PED}$) and relatedness (AR) coefficients from 1984 to 2019; (**B**) pedigree completeness index in the whole (WP) and reference (RF) populations.

The evolution of $F_{PED}$ and AR over time is shown in Figure 1A. The relatedness coefficient is inversely related to genetic diversity and can be used as an indicator of inbreeding in the long term. When AR is greater than $F_{PED}$ in the population, the mating between relatives is more frequent, and in general, when AR tends to approach zero, the genetic diversity increases. Therefore, when selecting top animals, it is important to consider the animals with lower AR values. The AR estimated in total and reference populations was 3.13 and 4.32%, respectively. The AR was always greater than $F_{PED}$, indicating that the frequency of mating between related animals was greater than the frequency of mating between unrelated individuals. It is also important to highlight that during the last 20 years, AR and $F_{PED}$ presented an increasing trend (Figure 1A). Thus, some efforts should be made to avoid genetic diversity erosion.

#### 3.1.2. Equivalent Complete Generations

The average equivalent complete generations were 2.9 in the total population and 4.7 in the reference population. The pedigree completeness index one generation ago was 70.9 and 95.9% in the total and reference populations, respectively. Tracing back five generations, the pedigree completeness index decreased to 31.5% in the total population and 54.3% in the reference population (Figure 1B). The pedigree completeness index is an important indicator of the $F_{PED}$ quality because it represents the harmonic mean of the parental genetic contributions, and it is zero if any parent is unknown regardless of how deep and complete the pedigree of the other parent is. The improvement in the pedigree completeness index seen at recent generations in the Mexican Romosinuano beef cattle population (Figure 1B) represents recent efforts that were put in place to improve the pedigree recording, and this strategy should continue.

#### 3.1.3. Generation Interval

The generation intervals computed using the four pathways of selection are presented in Table 1. The average generation intervals for the total and the reference populations were similar, with values of 6.25 and 6.52 y, respectively.

#### 3.1.4. Realized Effective Population Size

The realized effective population size based on the individual increase in $F_{PED}$ was 86.44 ± 14.69, and the individual increase in $F_{PED}$ was 0.578%. The $N_{e}$ is above the threshold of 50 recommended to maintain the population’s genetic diversity at an acceptable level [31].

#### 3.1.5. Probabilities of Gene Origin

The $f_{e}$ was 71 and 75, while the $f_{a}$ was 31 and 30, in the total and reference populations, respectively. A total of 10 and 11 ancestors explained 50% of the genetic diversity in the total and reference populations, respectively. The $f_{a}$/$f_{e}$ ratio was 0.44 in the total population, and it was 0.40 in the reference population, indicating the existence of genetic bottlenecks and loss of genetic diversity in the Mexican Romosinuano beef cattle population. The genetic bottlenecks were narrower in recent years, which agrees with a recent steeper increase in $F_{PED}$ (Figure 1A).

Ramírez-Valverde et al. [32] studied the genetic diversity using pedigree data in six Mexican beef cattle populations of cosmopolitan breeds and reported similar parameters for some breeds, compared to the Mexican Romosinuano beef cattle population presented here. In their study, the progeny number per sire and dam ranged from 6 to 31 and from 1.8 to 2.7, respectively. The authors reported $F_{PED}$ ranging from 0.9 to 4.2%, and AR from 0.3 to 6.5%, with equivalent complete generations varying from 2.03 to 7.51, generation interval from 5.1 to 7.2 y, and the effective population size from 24 to 192.

Based on the pedigree analysis, the $F_{PED}$ in the Mexican Romosinuano beef cattle population seems to be low. However, an increasing trend was observed, especially in recent years. Overall, the pedigree completeness index was low, with evident improvements in recent years. The generation interval was 6.52 years in the more recent generation, and this value is similar to other beef cattle populations Ramírez-Valverde et al. [32]. The effective population size (86.44) was close to the recommended minimum (50) to maintain genetic diversity at acceptable levels. Finally, genetic bottlenecks were present and narrower in the last generation.

### 3.2. Genomic Analyses

#### 3.2.1. Inbreeding Coefficients

Inbreeding in itself is neither good nor bad because it reflects homozygosity accumulation. In fact, the primary objective of genetic selection is to increase the frequency of favorable variants, and it can happen in homozygous or heterozygous genotypes. The inbreeding coefficient cannot differentiate between the accumulation of homozygosity of favorable alleles and the accumulation of homozygosity for neutral or deleterious alleles; therefore, it is an imperfect metric of the recessive load of an individual [33].

One way to better understand this situation is by looking at the inbreeding age, measured through the length of ROH segments. This is possible because haplotypes are broken by recombination over time; therefore, short segments were more likely to originate from a more distant origin (i.e., in old generations; ancient inbreeding) [34,35]. On the other hand, recent inbreeding produces long ROH segments with deleterious variants segregating for less time and not still filtered out by purging events yet [33]. Thus, long ROH (recent inbreeding) segments are a better metric of the recessive load of a given individual [36].

Summary statistics of ROH identified across different length classes are reported in Table 2. Across all genotyped animals, a total of 3943 ROH were found; the 1–2 Mb class was the most abundant, accounting for 48.4% of total ROH regions. Only 48 ROH were detected in the upper length class (>16 Mb), and these long regions were identified in 27 different animals; one of these animals showed six long regions and was the animal with the greatest $F_{ROH}$ (20.74%). The additive relationship between the parents of this cow was 13.3%, and they shared eight common ancestors across five generations in the pedigree. Of those eight commons ancestors, three of them were part of the group of the 10 ancestors, explaining 50% of the genetic variability in the population. Altogether, these three ancestors explained 25% of the genetic variation in the population. The $F_{GRM}$ of this cow was 17.34%, and it had the greatest $F_{GRM}$ among the group of genotyped animals. However, the $F_{PED}$ of the cow was not the largest among all the genotyped animals. In fact, the $F_{PED}$ was 6.66%, which is considerably lower than the other inbreeding estimates. This highlights the value of genomic information as a more accurate source of information.

The shortest and the longest ROH segments were 1.0 and 36.0 Mb, respectively. The greatest number of ROH per chromosome was found on BTA1 (321), whereas the lowest number was found on BTA28 (50). At the genome-wide level, a large proportion of ROH in all autosomes was in the shortest class (<2 Mb).

Although recent inbreeding is a useful measure to evaluate recessive load, the separation between ancient and recent inbreeding is still an active research topic and remains unclear. Maltecca et al. [33] reported that inbreeding depression was greater for more recent inbreeding than older inbreeding in a Holstein population. Using also Holstein cattle data, Makanjuola et al. [37] carried out a study splitting inbreeding into age classes and concluded that recent inbreeding had more detrimental effects, whereas ancient inbreeding caused even favorable effects. These authors reported a loss of −1.56 kg in 305-d protein yield associated with an increase of 1% in recent inbreeding (ROH > 4 Mb). Conversely, a gain of 1.33 kg in the same trait was associated with an increase of 1% in ancient inbreeding (ROH < 4 Mb). A recent research study conducted in beef cattle by Sumreddee et al. [38] demonstrated that although the recessive load is expected to be larger in longer ROH segments, short ROH segments (<5 Mb) can still harbor some deleterious mutations with substantial joint effects on some traits.

In the Mexican Romosinuano beef cattle population, 23.8% of the ROH segments were >4 Mb (Table 2) and potentially represented the recessive load in this population. However, it is important to recognize the small sample size and the necessity to expand the study. More genotyped animals are required to conduct a more comprehensive investigation of ROH regions in this breed.

The average ROH length ($L_{ROH}$, 3.29 ± 3.19 Mb) found in this study was slightly greater than values reported for other small cattle populations but smaller than values estimated in cosmopolitan breeds. Cesarani et al. [39], using the same ROH settings (i.e., minimum 15 SNP, 1 Mb of minimum length and 0 missing and heterozygotes allowed), reported the mean ROH lengths of 2.3 ± 1.8, 2.6 ± 2.3, and 2.4 ± 2.0 Mb for Modicana, Sardo-Bruna, and Sardo-Modicana breeds, respectively. Marras et al. [40] reported $L_{ROH}$ values of 3.9 and 3.6 for Brown Swiss and Holstein. The same authors reported an $L_{ROH}$ of 1.9 (i.e., almost half of the values found in this study) for the Piedmontese cattle breed. The $L_{ROH}$ observed in this study was relatively smaller than that of an inbred line of the Hereford cattle population (6.83 ± 4.45 Mb), as reported by Sumreddee et al. [19].

The length of ROH is a crucial parameter because it is associated with inbreeding events. Long ROH can be found when the mating between relatives occurred recently, whereas short ROH are signs of past events [41]. Gibson et al. [42] and Bosse et al. [43] reported that long homozygote segments are likely to be identical by descent. The results in the present study indicate that the majority of autozygous (ROH) segments (1–2 Mb class) identified in this population originated approximately 25 to 50 generations ago, assuming 1 cM equals 1 Mb [44].

On average, 53.97 ± 17.15 ROH were found per animal, with this value ranging from 18 to 102. This value is lower than those reported in the literature for cosmopolitan breeds; Marras et al. [40] found 81.7 ROH per animal in Holstein, whereas Ferencakovic et al. [45] reported 98.9 ± 10.2 ROH per animal in Brown Swiss. However, the ROH per animal presented in this study is greater than the values reported for small populations, such as Polish Red (46.4 ± 9.8; Szmatoła et al. [46]). A similar value (54.0 ± 7.2) was estimated for the Piedmontese cattle breed by Marras et al. [40].

The average total ROH length per animal ($S_{ROH}$) was 180.45 ± 92.40 Mb. Compared to $S_{ROH}$ found in other studies with cosmopolitan breeds, the $S_{ROH}$ found in the present study had an intermediate value. For instance, Szmatoła et al. [46] reported $S_{ROH}$ of 290.6 ± 67.2, 142.8 ± 67.4, and 180.5 ± 79.9 Mb for Holstein, Polish Red, and Limousin, respectively. Larger $S_{ROH}$ per animal were reported in the literature for Brow Swiss (371 Mb) and Holstein (297 Mb) by Marras et al. [40]. The same authors reported smaller values for the Piedmontese cattle (106 Mb).

**Figure 2:**
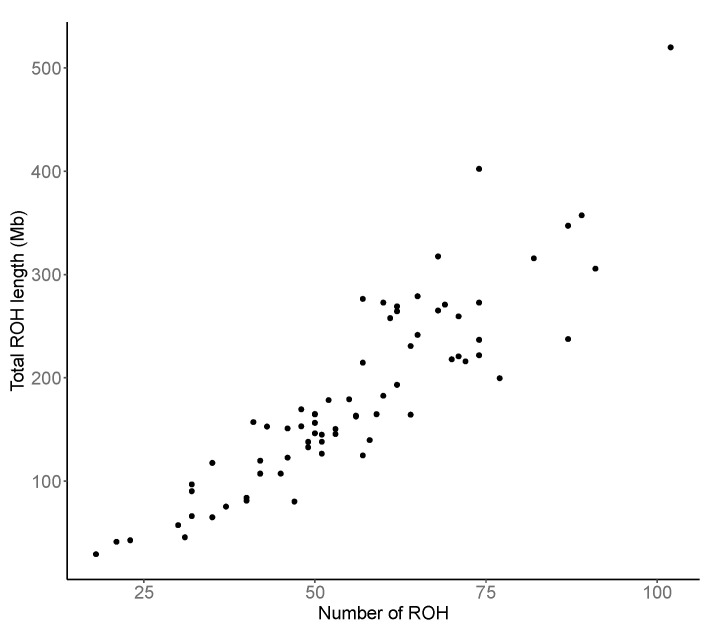
Relationship between the number of runs of homozygosity (ROH) found in each individual and their total length (Mb).

In general, animals with a larger ROH number tend to have a greater total length of ROH segments regardless of the length of single ROH regions (Figure 2). The correlation between the number of ROH identified and the total ROH length was 0.9, meaning that the more ROH regions, the larger is the total ROH length. A Similar result (correlation = 0.78) was recently published by Cesarani et al. [39] in European Simmental bulls.

**Figure 3:**
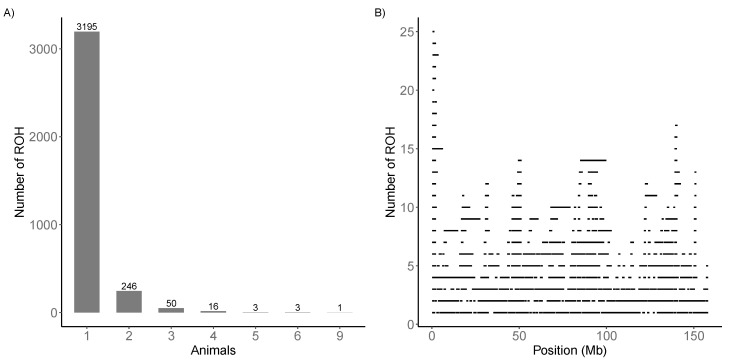
(**A**) Specific runs of homozygosity (ROH) distribution. (**B**) Stacked runs in chromosome 1.

A total of 319 regions were shared (specific ROH) by at least two animals (Figure 3A). The most shared ROH region was found in nine animals on chromosome 1, located between 0.13 and 2.36 Mb. The similarity among animals in this region was also confirmed by the plot of stacked runs (Figure 3B).

Inbreeding coefficients based on pedigree and genomic information for the 71 genotyped animals are shown in Table 3. Considering all detected ROH (i.e., >1 Mb), the average $F_{ROH}$ was 7.28%, and it decreased as the minimum length of ROH increased. This result reflects a decreased number of ROH identified as shorter ROH segments were excluded when longer ROH classes were considered. The average $F_{ROH}$ was 1.44% considering the >16 Mb class (Table 3), and this can be attributed to the fact that only a few ROH segments larger than 16 Mb were found. The number of inbred animals in the other ROH classes varied. The total number of inbred animals was 71 for $F_{ROH}$ > 1 Mb or > 2 Mb, 68 for $F_{ROH}$ > 4 Mb or > 8 Mb, and 27 for $F_{ROH}$ > 16 Mb. The largest $F_{ROH}$ value was observed for one animal with 20.74% of its genome covered by ROH (>1 Mb class).

The level of ROH based inbreeding varies in different populations. For instance, Ferencakovic et al. [47] reported an $F_{ROH}$ value of 9.0 ± 2.2% for Austrian Simmental bulls, and Szmatoła et al. [46] published an $F_{ROH}$ of 11.6 ± 2.6% for Holstein, 8.1 ± 3.9% for Simmental, 7.2 ± 3.2% for Limousin, and 5.7 ± 2.6% for Polish Red cattle.

**Figure 4:**
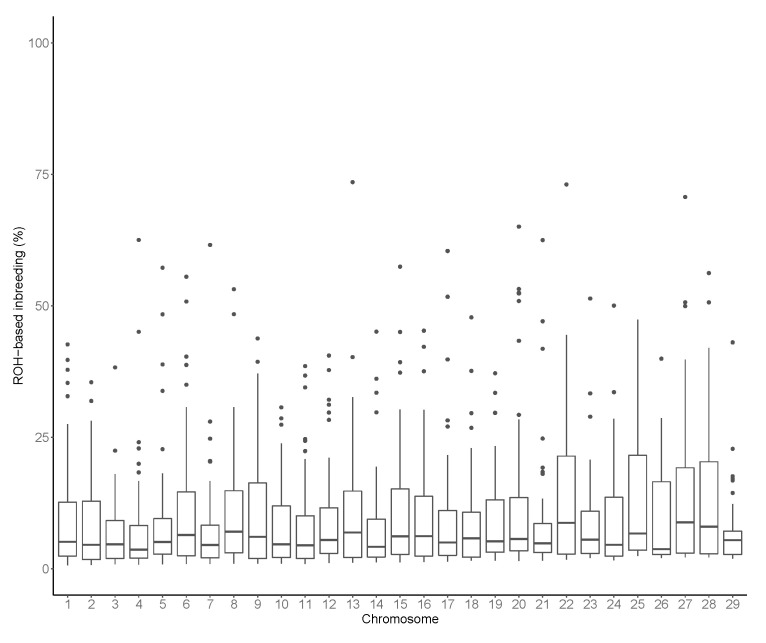
Runs of homozygosity (ROH) based inbreeding in each considered autosome.

The $F_{ROH}$ varied across the autosomes (Figure 4), with the smallest estimates found in BTA3 (6.55%) and the largest value on BTA27 (14.75%). Variation in $F_{ROH}$ across chromosomes was also reported in other cattle breeds (e.g., Sumreddee et al. [19]). As stated by Meyermans et al. [48], ROH became the state-of-the-art method for inbreeding assessment during the last decade. Thus, several studies focused on the $F_{ROH}$ estimation in several livestock species.

Table 4 shows the correlation between inbreeding coefficients for the group of genotyped animals. The correlations between $F_{PED}$ and $F_{GRM}$ or $F_{ROH}$ were non-significant (*r* = −0.25 to 0.31). This and the difference in estimates based on pedigree and genotypes highlights the importance of using genomic information in the assessment of genetic diversity. The use of different measures may lead to different conclusions.

The correlations among $F_{ROH}$ estimates based on different ROH lengths were high (*r* = 0.69 to 1.00) and significant (*p* < 0.05), whereas the correlations among $F_{ROH}$ estimates and $F_{GRM}$ were moderate and non-significant (*r* = 0.44 to 0.58). Although non-significant, the correlations among $F_{GRM}$ and $F_{ROH}$ declined as the ROH length increased, which was expected because $F_{GRM}$ captures all homozygous segments in the genome, which is not the case for ROH. This would make ROH a better approach to differentiate old and recent inbreeding. Contrary to $F_{PED}$, inbreeding coefficients estimated using genomic information do not depend on the knowledge of relatives, and therefore, these estimates are not biased by missing or incorrect pedigrees.

Previous studies have reported moderate correlations among $F_{GRM}$ and $F_{ROH}$. For example, the correlation between these inbreeding estimates ranged from 0.62 to 0.65 in dairy cattle breeds [49], and it was 0.56 for the Hereford breed [19].

#### 3.2.2. Linkage Disequilibrium and Effective Population Size

**Figure 5:**
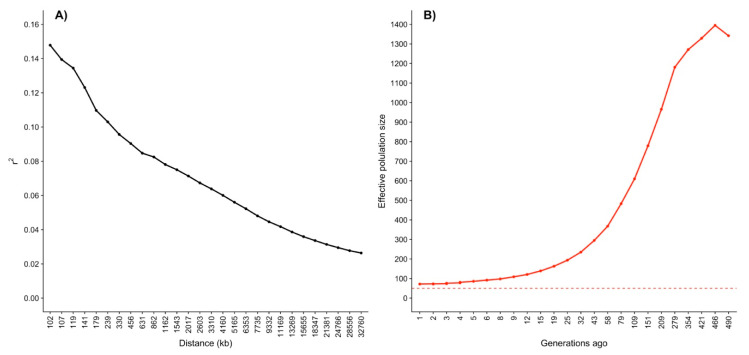
(**A**) Linkage disequilibrium estimated through the correlation between pairs of loci ($r^{2}$) over distances between markers; (**B**) effective population size trend from 1 to 490 generations ago, the dashed line represents the threshold of 50 proposed as the minimal values to maintain genetic diversity in acceptable levels.

The $r^{2}$ trend over distances between SNP from 100 to 35,000 kb is presented in Figure 5A. The $r^{2}$ declined from 0.147 to 0.026 when the distance between SNP pairs increased from 102 kb to 32,760 kb. Such observed decay in LD as the distance between markers increases is a typical trend found in other populations [50,51]. Decay in $r^{2}$ level was also studied for Romosinuano beef cattle in Colombia by Bejarano et al. [52]. These authors analyzed LD decay up to 200 and 500 kb and reported larger $r^{2}$ values than those found for the same distances between SNP in the present study. The observed difference is most likely due to differences in the genetic architecture between the populations. In general, populations under stronger selection have greater $r^{2}$ levels.

Linkage disequilibrium is a good indicator of associations between alleles of two or more loci. Larger LD values are usually estimated in homogeneous or closed populations because of loci inheritance from common ancestors. Thus, LD is greater in selected populations. Since the selection pressure is related to the Ne, LD can be utilized to estimate Ne [53,54]. The Ne 490 generations ago was 1342 animals, 109 generations ago it was 610, and it was reduced 1 generation ago to 72 (Figure 5B). The latter was a similar value to the Ne of 86.44 ± 14.6 estimated based on the pedigree. Indeed, the Ne estimated using genomic information fell inside the 95% confidence interval (57.82–115.05) for the pedigree-based estimate of Ne. The reduction in Ne moving from the past generations to the recent generations is a common feature of livestock [50,54] and implies a reduction in genetic diversity. It is important to highlight that the Ne estimated 1 generation ago was close to the critical point of 50 animals proposed as the threshold to have acceptable levels of genetic diversity [31]. Therefore, an appropriate mating design is needed to maintain or even increase the genetic diversity within the Mexican Romosinuano beef cattle breed.

### 3.3. Selection Signatures

A total of 2390 ROHet were detected, of which 29% had a length < 1 Mb, 64% had a length from 1 to 2 Mb, and only 7% were longer than 2 Mb. The minimum number of ROHet per animal was 25, the maximum was 68, and the mean number was 47.7.

**Figure 6:**
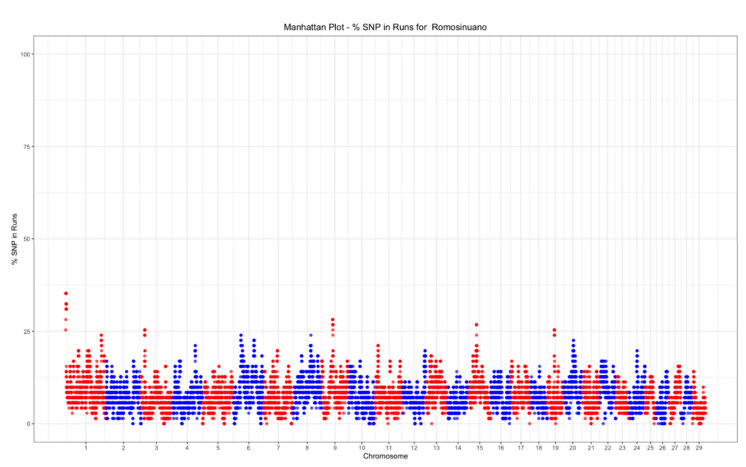
Manhattan plot of the SNP frequency within runs of homozygosity by autosome in the Mexican Romosinuano beef cattle population.

**Figure 7:**
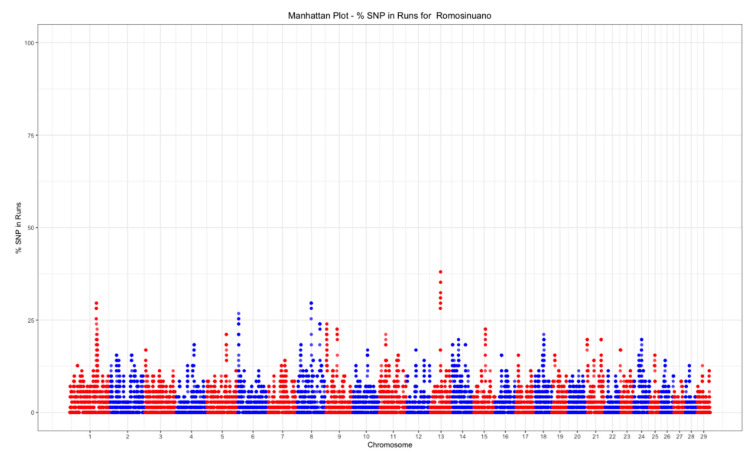
Manhattan plot of the SNP frequency within runs of heterozygosity by autosome in the Mexican Romosinuano beef cattle population.

The top 0.01% threshold for ROH was 32.39%, while it was 28.16% for ROHet. Only one ROH island was found on chromosome 1 (Figure 6), while three ROHet islands were found on chromosomes 1, 8, and 13 (Figure 7).

Table 5 presents the chromosome position, the start and end of islands on the chromosomes, the number of SNP, and genes found in the ROH and ROHet islands. Overall, 22 genes were found on both ROH and ROHet islands. The RCAN1 gene was found in the ROH island, and it plays an important role in the proliferation of smooth muscle cells. This gene was previously reported to be associated with the ribeye area in Nellore [55] and Wagyu cattle [56]. The TRNA-CCC gene was reported to be associated with immune-response processes (somatic cell score) and membrane transport in dairy cows and buffalos [57,58]. The KCNE1 and KCNE2 genes were found to be related to growth traits, such as weaning weight in Blanco Orejinegro beef cattle in Colombia, as reported by Londoño-Gil et al. [59].

Among genes found in ROHet islands, the TLEA4 gene is crucial for the function of the mammary gland and has previously been reported in Angus cattle by Devani et al. [60]. Additionally, several genes (CFAP61, RALGAPA2, INSM1, and KIZ) were located in a region associated with feed intake, conformation, weight, reproductive traits, and milk production in cattle, as demonstrated by Pitt et al. [61].

### 3.4. Inbreeding Depression

Inbreeding depression is a negative consequence of a high rate of inbreeding and can reduce the population’s average performance due to the increased frequency of homozygous alleles that are unfavorable or deleterious [62].

The effects of inbreeding on birth and weaning weights are presented in Table 6. Significant (*p* < 0.003) effects of inbreeding were found only in the first group of animals (i.e., all the animals with phenotypes). A 1% increase in inbreeding ($F_{PED}$) decreased birth weight by 0.103 kg and weaning weight by 0.685 kg. Similar results were reported by [19] in a research study that used data from the highly inbred line 1 of Hereford cattle in the U.S. In their research, reductions of 0.05 kg in birth weight and 1.2 kg in weaning weight were associated with an increase in $F_{PED}$ of 1%. In the same study, the inbreeding depression was stronger for $F_{ROH}$ and $F_{GRM}$ than for $F_{PED}$. In the present study, within the group of animals with genotypes and phenotypes, inbreeding depression was non-significant (*p* ≥ 0.079) for both pedigree and genomic inbreeding ($F_{GRM}$ or $F_{ROH}$). These non-significant inbreeding effects are likely attributed to a lack of statistical power due to the small sample size rather than the actual effect of inbreeding on phenotypes.

Based on a meta-analysis conducted on 57 studies and seven livestock species considering a wide variety of traits under selection, Leroy [63] estimated that inbreeding depression corresponded to an average decrease of 0.137% in the mean of a trait per 1% increase in inbreeding.

Several recent studies investigated the association between production or functional traits and different inbreeding measures ($F_{PED}$, $F_{GRM}$, and $F_{ROH}$) [19,36,37]. Martikainen et al. [64] identified that genotypes with high values of $F_{ROH}$ had significant unfavorable effects on Finnish Ayrshire cattle fertility traits. Cesarani et al. [39] investigated the effect of the presence/absence of particular ROH on estimated breeding values for milk, fat, and protein in European Simmental bulls and reported that some ROH, shared by at least 20 animals, showed significant adverse effects on all the traits.

A selection strategy to maximize genetic progress and constrain the inbreeding rate should be applied in the Romosinuano Mexican beef cattle population. The optimal genetic contributions method developed by Meuwissen [65] requires the estimated breeding values and relationships among selection candidates. Therefore, it could be easy to implement in this population. The optimal contribution method yields the genetic contributions of selected candidates to the next generations in terms of progeny number per candidate while constraining the average relationship to a given level among selection candidates and, thus, the inbreeding coefficient in the next generation.

## 4. Conclusions

In the Mexican Romosinuano beef cattle population, the inbreeding coefficient was larger when evaluated based on runs of homozygosity (7.28%) than when estimated based on the pedigree (2.98%) and the genomic relationship matrix (2.98%). Runs of homozygosity of length <4 Mb were more abundant in the autosomal genome. The correlation between $F_{PED}$ and $F_{GRM}$ was −0.25, and the correlations among $F_{PED}$ and $F_{ROH}$ of different length classes were low and ranged from 0.16 to 0.31. The correlations among $F_{GRM}$ and $F_{ROH}$ of different length classes were moderate and ranged from 0.44 to 0.58. Genetic bottlenecks were found in the population, and the effective population size presented an important reduction from 610 (109 generations ago) to 72 animals (1 generation ago), which approached the critical value recommended to maintain the genetic diversity at an acceptable level. The reduction in effective population size implies a decline in genetic diversity due to the intensive use of few individuals as parents of the next generations. Selection signatures were detected through highly homozygous and heterozygous regions related to immune response, growth, and reproductive traits. Inbreeding depression analyses showed that a 1% increase in an animal’s pedigree-based inbreeding coefficient resulted in a significant decrease in birth (−0.103 kg) and weaning (−0.685 kg) weights. A strategy, such as optimum genetic contributions to maximize selection response and manage the long-term genetic variability and inbreeding could lead to design more sustainable breeding programs for the Mexican Romosinuano beef cattle breed.

## Figures and Tables

**Table 1:** Estimates (±SE) of generation interval (GI) for the four selection paths in Mexican Romosinuano beef cattle.

Path of Selection	*n*	GI ± SE (y)
Total population		
Sire–sire	1211	6.73 ± 0.11
Sire–dam	2428	6.27 ± 0.06
Dam–sire	1270	6.49 ± 0.09
Dam–dam	2006	5.76 ± 0.07
Total	6915	6.25 ± 0.04
Reference population		
Sire–sire	459	6.21 ± 0.15
Sire–dam	557	6.12 ± 0.16
Dam–sire	470	7.09 ± 0.17
Dam–dam	544	6.71 ± 0.18
Total	2030	6.52 ± 0.08

**Table 2:** Number, mean length, and number of single-nucleotide polymorphisms (SNP) of runs of homozygosity in different length classes.

ROH Length Class	Number of ROH (%)	Mean ± SD	
		Length, Mb	SNP
1–2 Mb	1907 (48.4)	1.43 ± 0.27	20 ± 7
2–4 Mb	1098 (27.8)	2.79 ± 0.57	35 ± 14
4–8 Mb	640 (16.2)	5.60 ± 1.10	68 ± 20
8–16 Mb	250 (6.3)	10.42 ± 2.00	124 ± 29
>16 Mb	48 (1.3)	20.34 ± 4.56	237 ± 70
Total	3943 (100)	329 ± 3.19	41 ± 39

**Table 3:** Estimates of inbreeding coefficients based on pedigree ($F_{PED}$) and genomic information ($F_{GRM}$ and $F_{ROH}$).

Statistic	Inbreeding Measure						
	$F_{PED}$	$F_{GRM}$	$F_{ROH>1Mb}$	$F_{ROH>2Mb}$	$F_{ROH>4Mb}$	$F_{ROH>8Mb}$	$F_{ROH>16Mb}$
Mean ± SD, %	2.98 ± 2.81	2.98 ± 4.01	7.28 ± 3.68	5.75 ± 3.54	4.20 ± 3.07	4.20 ± 3.07	1.44 ± 1.05
Minimum, %	0.00	−3.35	1.17	0.17	0.17	0.17	0.64
Maximum, %	14.28	17.34	20.74	18.89	16.39	16.39	4.36

**Table 4:** Pearson’s correlations (above diagonal) among genomic ($F_{GRM}$), pedigree ($F_{PED}$) and Runs of Homozygosity ($F_{ROH}$) based inbreeding coefficients, significance values ^1^ are reported below the diagonal.

Inbreeding Measure		$F_{GRM}$	$F_{PED}$	$F_{ROH}$				
				>1 Mb	>2 Mb	>4 Mb	>8 Mb	>16 Mb
	$F_{GRM}$		−0.25	0.58	0.51	0.46	0.46	0.44
	$F_{PED}$	n.s.		0.16	0.24	0.24	0.24	0.31
$F_{ROH}$	>1 Mb	n.s.	n.s.		0.99	0.96	0.96	0.69
	>2 Mb	n.s.	n.s.	***		0.98	0.98	0.73
	>4 Mb	n.s.	n.s.	***	***		1.00	0.81
	>8 Mb	n.s.	n.s.	***	***	***		0.81
	>16 Mb	n.s.	n.s.	*	*	**	**	

^1^ n.s. = non-significant; * = *p* < 0.05; ** = *p* < 0.01; *** = *p* < 0.001.

**Table 5:** Description of the runs of homozygosity (ROH) and heterozygosity (ROHet) islands found in the Mexican Romosinuano beef cattle population’s autosomal genome.

Island Type	BTA	Start (bp)	End (bp)	n SNP	Genes ^1^
*ROH* island	1	39,3248	139,7493	13	*LOC112447010, LOC100138661, LOC1077131365, LOC618212, LOC112447011, LOC104971400, LOC787710, LOC112447083, CLIC6, RCAN1, LOC618840, TRNA-CCC, KCNE1, LOC101904627, LOC10190344, C1H2orf140, SMIM11A, KCNE2, LOC112446832, LOC112447088, LOC107132170, MRPS6*
ROHet island	1	103,788,356	104,344,681	9	*LOC112448262, LOC 112448289*
	8	55,663,248	56,244,907	13	*TLE4, LOC112447804, LOC104972906, LOC112447806, LOC104969368, LOC107132695*
	13	39,826,900	41,023,312	17	*CFAP61, INSM1, RALGAPA2, LOC112449374, LOC104973781, LOC112449451, KIZ, LOC100140493, XRN2, NKX2-4, LOC112449289, NKX2-2, LOC614124, PAX1*

^1^ Non-annotated loci are indicated with the prefix LOC.

**Table 6:** Estimates of the regression coefficients of pedigree inbreeding $\left(F_{PED}\right)$, genomic inbreeding based on the genomic relationship matrix $\left(F_{GRM}\right)$ and based on runs of homozygosity $\left(F_{ROH}\right)$ on birth weight and weaning weight.

Group/Inbreeding Coefficient	Birth Weight			Weaning Weight		
	Estimate ± SE	*p* value	*n*	Estimate ± SE	*p* value	*n*
With phenotypes						
$F_{PED}$	−0.103 ± 0.032	0.001	1328	−0.685 ± 0.229	0.003	690
With phenotypes and genotyped						
$F_{PED}$	−0.044 ± 0.218	0.841	51	−2.021 ± 2.222	0.373	37
$F_{GRM}$	−0.183 ± 0.170	0.291	51	−2.771 ± 1.507	0.079	37
$F_{ROH}$	−0.064 ± 0.047	0.185	51	−0.444 ± 0.349	0.212	37

## Data Availability

The dataset is not publicly available since it belongs to *“Asociación Mexicana de Criadores de Ganado Romosinuano y Lechero Tropical”* and is treated as confidential information.
